# Cooling Methods Used to Manage Heat-Related Illness in Dogs Presented to Primary Care Veterinary Practices during 2016–2018 in the UK

**DOI:** 10.3390/vetsci10070465

**Published:** 2023-07-15

**Authors:** Emily J. Hall, Anne J. Carter, Jude Bradbury, Sian Beard, Sophie Gilbert, Dominic Barfield, Dan G. O’Neill

**Affiliations:** 1 Department of Clinical Science and Services, The Royal Veterinary College, Hawkshead Lane, North Mymms, Hatfield, Herts AL9 7TA, UK; jubradbury@rvc.ac.uk (J.B.); dbarfield@rvc.ac.uk (D.B.); 2 Animal and Veterinary Sciences, Scottish Rural Colleges, West Mains Road, Edinburgh EH9 3JG, UK; anne.carter@sruc.ac.uk; 3 Pathobiology and Population Sciences, The Royal Veterinary College, Hawkshead Lane, North Mymms, Hatfield, Herts AL9 7TA, UK; sbeard@rvc.ac.uk (S.B.); doneill@rvc.ac.uk (D.G.O.); 4 Vets Now, Penguin House, Castle Riggs, Dunfermline KY11 8SG, UK; sophie.gilbert@vets-now.com

**Keywords:** canine hyperthermia, canine heat-related illness, active cooling, cold-water immersion, evaporative cooling, cooling dogs, VetCompass

## Abstract

**Simple Summary:**

Heat-related illness (HRI) is a potentially fatal disorder that can occur in dogs following exercise or exposure to hot environments. While many risk factors can affect the probability of HRI occurring, the priority for treating dogs with HRI is early and rapid reduction in their core body temperature to limit disease progression. Cold-water immersion (conductive cooling) and water spray with air movement (evaporative cooling) are the recommended treatments for dogs with HRI, with cooling attempts in dogs with HRI being strongly advised to take place prior to transportation for veterinary care. This retrospective study of cooling methods used in UK veterinary practices during 2016–2018 reports that just 21.70% of dogs were cooled prior to transport, and only 23.97% of dogs were cooled using the recommended methods between 2016 and 2018. These results highlight the need for clearer messaging to the public and to veterinary professionals on optimal management of dogs with HRI, with priority on promoting wider sharing of the advice to “cool first, transport second”.

**Abstract:**

The management of heat-related illness (HRI) in dogs has received limited attention in the veterinary literature, especially regarding effective cooling methods. Guidelines published in 2016 for prehospital management of dogs with HRI advised “cool first, transport second”, and recommended using cold-water immersion and evaporative cooling (water application with air movement) as the optimal approaches to reduce the patient’s temperature. The current retrospective cross-sectional observation study analysed electronic patient records from the VetCompass programme to describe the cooling methods used in dogs with HRI presented to primary care veterinary practices during 2016–2018. Of 623 HRI events identified, 341 (54.74%, 95% CI 50.81–58.60%) included information on cooling in their clinical record. Of these, 74/341 (21.70%, 95% CI 17.65–26.38%) were cooled prior to transport for veterinary care. Overall, 23.97% (95% CI 19.24–29.44%) were cooled using one of the two recommended cooling methods, whilst the most common cooling method recorded was the application of wet towels (51.31%, 95% CI 45.34–57.24%). Canine cooling guidance and messaging in both the public and veterinary sectors requires urgent review to ensure that the most effective cooling methods are promoted because delays to canine temperature reduction worsen patient outcomes.

## 1. Introduction

Heat-related illness (HRI) occurs when an animal’s body temperature elevates to the point that their thermoregulatory abilities are saturated and the animal cannot effectively cool themselves to a stable level [1,2]. This results in harmful hyperthermia that causes biochemical derangements and tissue damage that may result in death if the hyperthermia is sustained [1,3,4]. There are two common triggers of HRI: environmental HRI triggered by inappropriate exposure to a hot environment (such as a hot vehicle, building or ambient conditions) and exertional HRI triggered by physical exertion typically in (but not limited to) hot conditions [1,4,5]. In the UK, exertional HRI accounts for 74.2% of HRI cases presented for veterinary treatment [5]. 

In medicine, HRI has been recognised for centuries [6]. Within studies reporting the epidemiology of HRI in humans, the importance of effective treatment is stressed [6,7,8,9]. Numerous studies have explored the effectiveness of various cooling methods [7,8,10,11,12,13], resulting in cold-water immersion in temperatures between 1.7 and 15.0 °C being recommended currently as the best practice method for cooling human athletes with HRI [12,14]. Cold-water immersion has been shown to reduce morbidity and mortality in human athletes and military personnel with exertional HRI cooled in real world situations [13,15], but these studies by their nature typically included healthy and physically fit people who were actively participating in intense physical activity [16]. These studies also typically included cases with exertional hyperthermia that had not yet progressed to severe HRI. Concerns have therefore been raised regarding recommending the use of cold-water immersion as a universal treatment recommendation for all grades of HRI if the patient is elderly, has neurological impairment or cardiovascular compromise [17]. These concerns commonly relate to patient discomfort due to shivering, hygiene due to the frequent occurrence of vomiting and diarrhoea in patients with HRI, and impaired access to patients should they require advanced cardiac monitoring or resuscitation [9,16]. For this reason, evaporative cooling—i.e., applying air movement whilst spraying water over the patient—is frequently recommended in a hospital setting as a preferred alternative to cold-water immersion [9,16,17]. A recent large-scale, prospective, multicentre study in Japan reported that human patients with severe HRI who were not actively cooled had 3.29 times the odds of death compared to patients who were actively cooled (using various cooling methods including cold-water immersion, evaporative cooling and internal cooling such as bladder or gastric lavage) [17]. Consequently, that study recommend active cooling should therefore be instigated in all patients with severe HRI [17]. 

In contrast, comparatively little attention has been given to effective management of HRI in dogs in the veterinary literature, especially regarding the comparative effectiveness of various cooling methods [18]. Immersion in 30.0 °C water has been shown to be effective in cooling hyperthermic military working dogs post-exercise [18]. The temperature of the water used in that study used a pragmatic approach to reflect the water likely to be available when dogs are deployed in hot working conditions [18]. An older laboratory-based study evaluated canine cooling in water from 1.0 to 25.0 °C, and reported that immersion in 15.0–16.0 °C water resulted in the most rapid cooling for conscious dogs with experimentally induced HRI, whilst comatose dogs cooled most quickly in 1.0–3.0 °C water [19]. However, that experimental study also reported that some dogs died immediately upon immersion into ice-cold-water, with the authors suggesting that increased vascular resistance caused by peripheral vasoconstriction may have triggered cardiovascular collapse [19]. This latter suggestion may have led to the recommendation frequently found in older veterinary texts and canine first aid advice to only cool dogs with “tepid” or “not cold” water and to avoid ice-water baths [20,21,22,23,24]. In consequence, some anecdotal reports exist of active decisions being made to delay cooling in dogs with HRI due to a lack of available “tepid” water (even when water was available but was deemed too cold). As the severity and negative outcomes of HRI in dogs are largely determined by the duration and degree of temperature elevation above 43.0 °C [25], any delay to cooling will worsen the outcome for the patient, and indeed cooling prior to presentation for veterinary treatment has been reported to improve canine survival [26]. Improved clarity in, and guidance relating to, cooling protocols for dogs with HRI is therefore urgently needed to ensure that cooling is always instigated in a timely and effective manner.

Despite extensive evidence to the contrary, another reason the advice that cold-water immersion should be avoided within management protocols for HRI has continued to be promoted within both medical and veterinary literature, is the belief and concern that peripheral vasoconstriction and shivering could cause further temperature elevations [3,12]. In contrast to this belief, Casa et al. go as far as to claim that “it is quite difficult, if not impossible, to kill an otherwise healthy [human] athlete experiencing [exertional heat stroke] if rapid cooling via cold/ice water immersion is implemented within a few minutes after collapse” [27]. Yet, in the United States, a disconnect has been noted between the sports medicine community’s recommendation of “cool first, transport second” for care of exertional HRI in people, compared to the emergency medical services where treatment protocols suggest not delaying transport to hospital by first cooling the patient [28]. Taking a clear position on the priority for early cooling, in March 2016, the American College of Veterinary Emergency and Critical Care’s Veterinary Committee on Trauma (Vet-COT) published best practice recommendations for prehospital care of dogs and cats that included the recommendation to actively cool patients with HRI prior to transporting the animal for emergency veterinary care [29]. Cold-water immersion was recommended by Vet-COT for young and healthy animals, whilst evaporative cooling (spraying the skin and coat with water in combination with air movement) was recommended for geriatric animals or those with comorbidities [29]. 

This study aimed to describe the cooling methods used to manage UK dogs presented to primary care veterinary practices with HRI during 2016–2018 to provide a benchmark on current canine cooling methods. An additional aim was to determine the degree to which Vet-COT recommendations regarding prioritising cooling before transport, and the use of cold-water immersion or evaporative cooling to deliver rapid cooling, were being followed. The study hypothesised that adherence to the Vet-COT guidelines increased over time following first publication in 2016.

## 2. Materials and Methods

This study continued the work previously reported by Hall et al. and used the same study population and datasets described in those studies [3,5]. The VetCompass programme provides access to de-identified veterinary patient records [30], including data from primary care, referral, charity and emergency care veterinary clinics in the UK. The current retrospective cross-sectional observation study reported on cooling methods used in the management of dogs presented with HRI, using a cohort of 945,543 dogs under primary veterinary care at 886 UK veterinary practices. From that cohort, the patient records of 856 dogs presented for management of HRI during 2016–2018 were reviewed to extract information relating to time between exposure to the HRI-triggering event and presentation for veterinary treatment, and on the patient cooling methods used. Only HRI events presented to the veterinary practice within 24 h of the triggering event were included in this review. Data extracted from the patient records included all methods of cooling applied to each patient, regardless of when and by whom these were performed (prehospital, or following presentation to the veterinary practice), and canine body temperature at presentation. Where cooling was stated in the patient records but the specific cooling method was not recorded, cooling method was categorised as ‘not specified’. Where no mention of cooling was identified in the patient records, these events were assigned as ‘no cooling method recorded’. For events where multiple methods of cooling were applied, every type of cooling action performed was extracted for each HRI event. For events where the type of cooling used was specified, the frequency and percentage (%) of use for each cooling method was calculated, with 95% confidence intervals (95% CI) calculated using the Wilson score interval using Epitools [31].

As the patient records available in the VetCompass programme were not recorded primarily for the purposes of research, the accuracy and level of detail included can vary [32]. Typed notes entered into the patient’s clinical record, some scanned documents (such as previous patient histories and emergency clinic histories) and charged items can be viewed, but not paper-based documents such a hospitalisation charts or anaesthetic record charts. For this reason, detailed notes relating to patient body temperature over time could not be consistently extracted if these had been recorded on paper-based systems. As the decision to cool a patient with HRI may be influenced by factors such as the clinical presentation of the dog, perceived severity of disease, availability of cooling methods and personal knowledge of the dog’s guardian or veterinary professional, it was not appropriate to carry out an analysis of potential relationships between cooling methods and HRI severity or outcome because there was limited information in the clinical records on these factors. Instead, a summary of the cooling data extracted is presented to benchmark the methods used in dogs under primary veterinary care during this period. This information can support future work that may specifically access these additional information sources or apply mixed-method approaches to provide greater depth of understanding on these issues. The mean, standard deviation (SD) and range of presenting body temperatures for dogs with a history of cooling versus no history of cooling is also presented. Body temperature had a normal distribution when assessed with the Kolmogorov–Smirnov test. The number and proportion of dogs (overall and by year) managed as per the Vet-COT prehospital care of dogs with HRI guidelines [29] is also reported, alongside the number of dogs cooled using the cooling methods recommended in those guidelines (cold-water immersion or evaporative cooling). To test the hypothesis that adherence to the Vet-COT guidelines increased over time following publication in 2016, the chi-squared test was used to compare the annual proportion of HRI cases cooled following those guidelines from 2016 to 2018. Statistical significance was set at the 5% level.

## 3. Results

### 3.1. Prehospital Cooling

**Figure 1:**
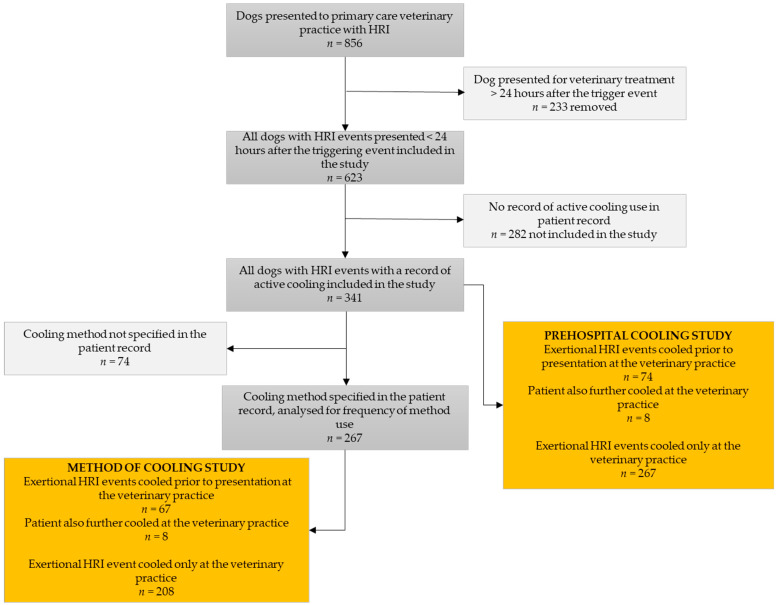
Flow chart illustrating the number of dogs with heat-related illness (HRI) events included in each part of the retrospective review.

The study cohort included 856 dogs presented for primary veterinary care during 2016–2018 with an HRI event in their patient record. Of these, 623 (72.78%, 95% CI 69.70–75.66%) events were presented for care within 24 h of the triggering event. No cooling actions were recorded in the patient record for 282/623 (45.26%, 95% CI 41.40–49.19%) of those HRI events (Figure 1). Of the 341 (54.74%, 95% CI 50.81–58.60%) dogs with active cooling recorded in their patient record, 74 (21.70%, 95% CI 17.65–26.38%) were cooled prior to presentation for veterinary treatment, of which 66 (89.19%, 95% CI 80.09–94.42%) received no further active cooling and 8 (10.81%, 95% CI 5.58–19.91%) did receive further active cooling from the veterinary practice. The remaining 267 (78.30%, 95% CI 73.62–82.35%) dogs were actively cooled only by veterinary professionals. The percentage of dogs cooled prior to presentation for veterinary treatment increased numerically across the three years of the study (Table 1) but this increase was not statistically significantly different by year (X^2^(2) = 1.66, *p* = 0.435).

### 3.2. Review of Cooling Methods Used

Of the 341 HRI events presented for treatment within 24 h of the triggering event with active cooling detailed in the patient record, the method of active cooling was not specified for 74 (21.70%, 95% CI 17.65–26.38%) events. Of the 267 dogs with the methods of active cooling specified in the patient record, 67 dogs were actively cooled prior to presentation, of which 17/67 (25.37%, 95% CI 16.49–36.93%) were actively cooled using a Vet-COT-recommended method (cold-water immersion or evaporative cooling). In comparison, 47/208 (22.60%, 95% CI 17.44–28.75%) dogs actively cooled by the veterinary practice were cooled using the recommended methods. Overall, 64/203 (23.97%, 95% CI 19.24–29.44%) dogs were cooled using a VetCOT-recommended method; although the percentage of dogs cooled using a recommended method reduced numerically across the three years of study (Table 2), this reduction was not statistically significantly different by year (X^2^(2) = 1.98, *p* = 0.372). The study hypothesis was therefore rejected.

Overall, the most frequently recorded cooling method used was application of wet towels (*n* = 137, 51.31%, 95% CI 45.34–57.24%) whilst the least frequently recorded method was application of a cold-water enema (*n* = 8, 3.00%, 95% CI 1.53–5.80%). The frequency of cooling methods employed both prior to presentation and by veterinary professionals is presented in Table 3. 

There were 515/623 (82.66%, 95% CI 79.49–85.43%) HRI events with a recorded canine body temperature at initial presentation for veterinary care; the mean body temperature at presentation was 39.9 °C (SD = 1.5 °C, range = 35.4 to 43.4 °C). Of the 14 dogs with a body temperature < 37.2 °C on presentation, six (42.9%, 95% CI 21.4–67.4%) were reported to have been cooled prior to presentation, while the remainder had no cooling method recorded in the patient history. The mean canine body temperature at presentation to the veterinary practice was 38.6 °C (SD = 1.5 °C, *n* = 66) for dogs cooled prior to presentation, 40.9 °C (SD = 1.1 °C, *n* = 267) for dogs cooled by the veterinary professionals, and 41.0 °C (SD = 0.9 °C, *n* = 8) for dogs cooled both prior to presentation and by veterinary professionals. The mean body temperature for dogs with no cooling method recorded in the patient history was 39.1 °C (SD = 1.0 °C, *n* = 282). 

## 4. Discussion

This study describes the cooling methods used to manage dogs with HRI under primary veterinary care in the UK during 2016–2018. Despite the 2016 Vet-COT recommendation to “cool first, transport second”, only 21.70% of the dogs with a record of cooling had been actively cooled prior to presentation for veterinary treatment. This is perhaps unsurprising, as the Vet-COT guidelines were published in March 2016 [29]. Dissemination of new guidelines and recommendations into the wider veterinary community often takes quite some time, and it is likely that the messages had not reached most primary care clinicians during the first year of the current study period. Whilst the percentage of HRI cases that received prehospital cooling did increase (albeit not significantly) from 19.76 in 2016 to 26.92% 2018, the cause behind any increase is not known and may be unrelated to the Vet-COT guidelines. Indeed, the percentage of dogs cooled using a recommended method reduced from 26.92% in 2016 to 17.91% in 2018, which could suggest that the Vet-COT guidelines were not responsible for the changes in cooling practices. It should also be noted that the Vet-COT guidelines were themselves based on the best available evidence [29], which at the time of publication consisted of largely human cooling studies [9,33]. Further research evaluating cooling methods for dogs with HRI is therefore urgently needed.

Whilst 45.26% of the dogs with HRI had no mention of cooling methods in the veterinary patient records, the mean presenting body temperature for those dogs was 39.1 °C. The Vet-COT guidelines recommend measuring canine body temperature in patients with suspected HRI and actively cooling if the temperature exceeds 41.1 °C; however, no source is provided in those guidelines to support this temperature cut-off for cooling action [29]. It is therefore possible that in those HRI cases with no mention of cooling in the patient record, the dog may have responded adequately to removal from the heat source or stopping exercise, and therefore active cooling was not required. It is also likely that some electronic patient records were simply missing information related to cooling due to the nature of time pressures in primary care practice, or that cooling methods used were recorded on paper hospitalisation charts, which were not accessible for analysis. These uncertainties highlight a well-recognised limitation of incompleteness when using patient records for research [32,34]. Of the cases with a record of cooling in the patient history, 21.70% did not specify the cooling method used. Again, this is likely a reflection of the nature of primary care records but could indicate that practitioners perceive cooling to be less noteworthy than other treatment modalities, such as pharmaceutical products or intravenous fluid therapy. 

The most frequently reported active cooling method used for dogs with HRI was the application of water-soaked towels (51.31%). There were also records of veterinary professionals using alcohol application to skin and cold-water enemas as cooling methods, despite recommendations against these methods in the literature; although, it must be noted that these recommendations come from review articles and not empirical studies evaluating these cooling methods. Alcohol application to the skin has been discouraged, as there is the potential for toxicity if it is absorbed from the skin, and there are concerns relating to flammability should defibrillation be required [10]. There are suggestions that cold-water enemas have the potential to worsen intestinal permeability disorders in patients with HRI [24,35], and studies evaluating the effectiveness of internal lavage cooling methods have reported external evaporative cooling (air movement whilst spraying the patient with water) to be more rapid, with fewer side effects [36]. The results of the current review of cooling methods used in UK primary care veterinary practices suggest that the cooling methods used during 2016–2018 did not reflect the Vet-COT recommendations for prehospital care of dogs with HRI [29]. 

In contrast to the human and equine literature, the application of water-soaked towels as a cooling technique appears frequently in online first aid recommendations for cooling dogs with HRI [23,24,37]. However, the cooling effects from application of water-soaked towels have been shown to be less rapid than cold-water immersion in humans [9], and ineffective in horses [38]. Empirical research evaluating the effectiveness of wet towels for cooling dogs is lacking, but clinical review articles have suggested that wet towels should be applied only to less-haired regions, such as the abdomen or inner thigh, because covering the entire dog in a towel (even if wet) could restrict air movement around the dog and reduce the potential for additional heat loss via evaporation and convection [20,24]. In the present study, it was not possible to determine the relative placement of towels on the body from the patient histories. However, the frequent use of water-soaked towels in primary care veterinary clinics may suggest that this cooling method is commonly selected over cold-water immersion or evaporative methods.

Just 22.60% of the dogs cooled by veterinary professionals were cooling using the Vet-COT-recommended methods (cold-water immersion or evaporative cooling) during the study period. It was not possible to determine the reasoning behind each cooling method selection in this study. Several factors could influence clinician decision making regarding cooling methods, including the patient’s clinical presentation, awareness of the Vet-COT guidelines, and accessibility of cooling methods. The availability of the resources required to perform cold-water immersion in veterinary practice may be limited, especially for large dogs, and concerns regarding hygiene and patient access may deter professionals from using water immersion. The use of cold-water immersion carries an additional risk of aspiration, particularly for dogs with respiratory compromise (such as breeds with brachycephaly) and unconscious dogs [22,24]. Patients undergoing cold-water immersion should be continuously monitored and the head supported where necessary to minimise risks of water aspiration. Most veterinary practices are likely to have access to the resources required to perform evaporative cooling (water spray and air movement from fans or air conditioning). Thus, the reasoning behind veterinary professionals’ choice of cooling method should be explored to determine how more effective cooling guidance can be promoted and to address barriers to its implementation where possible. 

Of the dogs with a record of active cooling in their patient record, just 21.70% of dogs that presented to veterinary practices for treatment of HRI had been cooled prior to travelling to the practice. Only eight of these dogs received further active cooling by veterinary professionals, suggesting the others had already been adequately cooled prior to presentation, as evidenced by the presenting body temperature records for dogs cooled only prior to presentation (mean = 38.6 °C) versus dogs cooled only by the veterinary practice (mean = 40.9 °C). It is not possible to identify the reasons behind cooling decisions made by these canine guardians from the data available in the current study, or to enumerate how many were advised to cool their dog prior to travelling by the veterinary practice staff versus independently making the decision to cool their dog based on their prior knowledge of first aid for managing HRI. However, the low proportion of dogs cooled prior to arrival at the veterinary practice suggests that the message to “cool first, transport second” needs to be more clearly communicated to both the public and the veterinary profession [29].

As previously noted, this study presents benchmark data summarising the cooling methods used for dogs presented for primary veterinary care during 2016–2018 in the UK but does not extend to presenting a comprehensive analysis of the effectiveness of those methods. Therefore, it is not possible to make recommendations on which are the most effective cooling methods based on the results of this study alone. Whilst further research is needed to fully evaluate the effectiveness and safety of the various cooling methods that can be applied to dogs with HRI, ethically this may be challenging [16]. A recent human study explored the relationship between patient body temperatures and survival for people hospitalised with severe HRI [39]. In the study, survivors had a greater reduction in body temperature during the first two hours of care compared to non-survivors, further supporting the clinical benefits of effective early cooling [39]. However, the non-survivor group had the lowest recorded body temperatures within 24 h of presentation, and organ damage was reduced when patient body temperature was between 38.5 and 40.0 °C by 30 min after presentation [39]. Both hypo- and hyperthermia were associated with adverse outcomes, leading the authors to recommend constant, accurate temperature monitoring when treating patients with HRI to avoid inadvertently causing harm through either over-aggressive or poorly effective active cooling [39]. It was not possible to extract detailed temperature monitoring records over time from the VetCompass programme in the present study, which prevented similar analysis for the dogs with HRI; however, this should be considered a priority for further research.

The VetCompass programme allows researchers to access the veterinary patient records of millions of animals, but it is important to note that these records were not generated for the primary purpose of research [32,40]. As a result, data may be missing from the patient records and the data present may be impacted by what each veterinary professional deemed pertinent to record. Whilst 45.26% of the HRI events included in this study had no cooling methods mentioned in the patient record, some of these dogs were examined after a short delay (if the dog’s condition deteriorated, for example), meaning they were not examined at the initial point of hyperthermia. It was not possible to determine precise timings from the patient records, and hand-written records such as hospitalisation sheets were inaccessible meaning only electronically entered temperature records could be accessed via VetCompass. 

Only veterinary practices in the UK were included in this study, meaning the results should be generalised with care to dogs outside of the UK. However, the benchmark results presented here for cooling in UK veterinary practices suggest that the current best practice recommendations regarding effective cooling methods were not aligned with the methods being used in primary care practice, and most dogs with HRI were not cooled prior to presentation for veterinary treatment. As extreme heat events are predicted to become more severe and frequent [41,42], veterinary professionals should consider reviewing the advice they provide to canine guardians regarding the immediate management of dogs with HRI, and also to audit their own protocols for cooling hyperthermic patients in the clinic. 

## 5. Conclusions

The cooling methods used to manage dogs with HRI presenting to primary care veterinary practices during 2016–2018 demonstrated poor alignment with the Vet-COT best practice recommendations for prehospital management of canine patients with HRI. Only 21.70% of the dogs treated for HRI had been recorded as cooled prior to arrival at the veterinary practice, highlighting the need for stronger communication of the message “cool first, transport second”. The most frequently used cooling method was the application of water-soaked towels (51.31%), despite strong evidence from both the human and equine literature that this method is less effective than cooling methods recommended in the Vet-COT guidelines (cold-water immersion or evaporative cooling). In conclusion, current messaging regarding the management of dogs with HRI requires urgent attention to dispel cooling myths; the message to “cool first, transport second” should be widely promoted, as delayed cooling will result in poorer clinical outcomes.

## Figures and Tables

**Table 1:** The number and percentage of dogs (with a record of cooling in their patient record) cooled prior to presentation for veterinary treatment of heat-related illness to UK primary care veterinary practices during 2016–2018 (*n* = 341).

Year of Study	Number of Dogs Cooled Prior to Presentation for Veterinary Treatment/Total Number of Dogs Cooled	Percentage of Dogs Cooled Prior to Presentation (95% Confidence Interval)
2016	33/167	19.76 (14.43–26.45)
2017	20/96	20.83 (13.91–30.00)
2018	21/78	26.92 (18.34–37.68)

**Table 2:** The number and percentage of dogs (with a cooling method specified in their patient record) cooled using a VetCOT-recommended cooling method by year, in dogs presented for treatment of heat-related illness to UK primary care veterinary practices during 2016–2018 (*n* = 267).

Year of Study	Number of Dogs Cooled Using a Recommended Method/Total Number of Dogs Cooled	Percentage of Dogs Cooled Using a Recommended Method (95% Confidence Interval)
2016	35/130	26.92 (20.04–35.13)
2017	17/70	24.29 (15.75–35.5)
2018	12/67	17.91 (10.55–28.75)

**Table 3:** The frequency and percentage (with 95% confidence intervals [95% CI]) of cooling methods used in the management of dogs with heat-related illness presented for primary veterinary care during 2016–2018 in the UK. Eight cases were cooled both prior to presentation, and by the veterinary practice.

	Cooling Method Used	Number of Cases Cooled with This Method (%, 95% CI of All Cooled Cases) *n* = 267	Number of Cases Cooled Prior to Presentation with This Method (%, 95% CI of Cases Cooled Prehospital) *n* = 67	Number of Cases Cooled with This Method by the Veterinary Practice (%, 95% CI of Veterinary Cooled Cases) *n* = 208
Vet-COT-recommended cooling method used:	Cold-water immersion or evaporative cooling	64 (23.97, 19.24–29.44)	17 (25.37, 16.49–36.93)	47 (22.60, 17.44–28.75)
	Cold-water immersion	45 (16.85, 12.84–21.81)	15 (22.39, 14.06–33.71)	30 (14.42, 10.29–19.84)
	Evaporative cooling	20 (7.49, 4.90–11.29)	2 (2.99, 0.82–10.25)	18 (8.65, 5.54–13.26)
Other cooling method used	Water-soaked towel	137 (51.31, 45.34–57.24)	26 (38.81, 28.05–50.78)	111 (53.37, 46.59–60.02)
	Air movement	84 (31.46, 26.19–37.26)	11 (16.42, 9.42–27.06)	73 (35.10, 28.93–41.80)
	Water spray (no air movement)	62 (23.22, 18.56–28.64)	19 (28.36, 18.97–40.09)	43 (20.67, 15.73–26.68)
	Ice/ice packs	46 (17.23, 13.17–22.21)	4 (5.97, 2.35–14.37)	42 (20.19, 15.3–26.17)
	Cold intravenous fluids	26 (9.74, 6.73–13.89)	0	26 (12.50, 8.67–17.69)
	Alcohol application	21 (7.87, 5.20–11.72)	0	21 (10.10, 6.70–14.94)
	Cold-water enema	8 (3.00, 1.53–5.80)		8 (3.85, 1.96–7.40)

## Data Availability

Supporting data are available via this link: https://rvc-repository.worktribe.com/output/1626117.
